# Evolutionary History of the HAP2/GCS1 Gene and Sexual Reproduction in Metazoans

**DOI:** 10.1371/journal.pone.0007680

**Published:** 2009-11-03

**Authors:** Robert E. Steele, Catherine E. Dana

**Affiliations:** Department of Biological Chemistry and the Developmental Biology Center, University of California Irvine, Irvine, California, United States of America; University of California, Riverside, United States of America

## Abstract

The HAP2/GCS1 gene first appeared in the common ancestor of plants, animals, and protists, and is required in the male gamete for fusion to the female gamete in the unicellular organisms *Chlamydomonas* and *Plasmodium*. We have identified a HAP2/GCS1 gene in the genome sequence of the sponge *Amphimedon queenslandica*. This finding provides a continuous evolutionary history of HAP2/GCS1 from unicellular organisms into the metazoan lineage. Divergent versions of the HAP2/GCS1 gene are also present in the genomes of some but not all arthropods. By examining the expression of the HAP2/GCS1 gene in the cnidarian *Hydra*, we have found the first evidence supporting the hypothesis that HAP2/GCS1 was used for male gamete fusion in the ancestor of extant metazoans and that it retains that function in modern cnidarians.

## Introduction

The evolutionary history of the machinery of sexual reproduction in metazoans has not yet been explored in detail. Particularly lacking is information from taxa that bridge the transition from unicellular organisms to metazoans. Choanoflagellates, the sister group to metazoans, have not been shown to undergo sexual reproduction [1]. Placozoans, which may be the most basal metazoans [2], have been seen to produce what appear to be eggs [3]. These apparent eggs cleave to yield up to 256 cells, but they fail to develop further [4]. Sperm production has not been demonstrated in placozoans [5]. Thus it is not clear to what extent the machinery of sexual reproduction was conserved when metazoans evolved from unicellular ancestors.

The increasing availability of genome sequences from diverse metazoan taxa promises to allow a tracing of this history by examining genes encoding proteins involved in sexual reproduction. One molecule of particular interest in this regard is the product of the HAP2/GCS1 gene, a gene that was originally identified because of its expression in male gametes of plants and unicellular eukaryotes and its requirement for fertilization in *Arabidopsis* [6], [7]. Recent studies have demonstrated that the HAP2/GCS1 protein functions in gamete membrane fusion in *Plasmodium* and *Chlamydomonas* [8], [9]. The presence of the HAP2/GCS1 gene in a choanoflagellate and in the cnidarians *Hydra* and *Nematostella* [8], [9] suggests a hypothesis in which HAP2/GCS1-mediated fusion of gametes was established in the ancestor of modern plants, protists, and animals and that it still plays such a role in some extant metazoans.

To test this hypothesis we looked for evidence of a HAP2/GCS1 gene in the genome of the sponge *Amphimedon queenslandica* and examined expression of the HAP2/GCS1 gene in *Hydra*. Our finding that the HAP2/GCS1 gene is present in sponges and is expressed in spermatogenic cells of *Hydra* supports the hypothesis.

## Results

### HAP2/GCS1 Is Present in Sponges but Absent from *Trichoplax*

**Figure 1:**
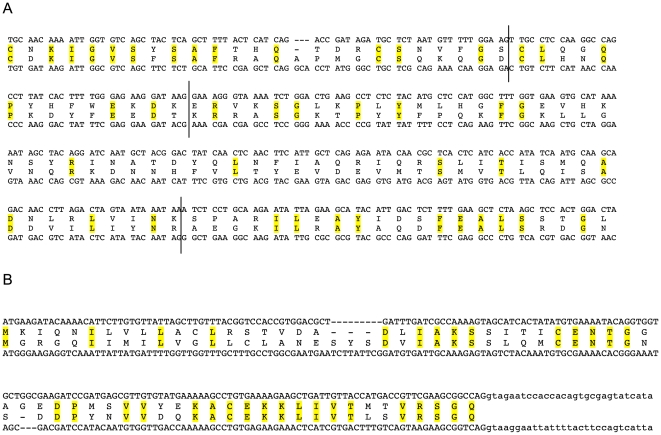
HAP2/GCS1 genes in the sponge *Amphimedon queenslandica* and the coral *Acropora palmata*. Panel A. Alignment of *Amphimedon* and *Nematostella* HAP2/GCS1 genomic sequences. Alignment of genomic sequences encoding a segment of the HAP2/GCS1 gene from the sponge *Amphimedon queenslandica* (upper sequence) and the sea anemone *Nematostella vectensis* (lower sequence) and are shown. Identical amino acids are highlighted in yellow. The three vertical lines indicate the locations of introns, which are in homologous locations in the two sequences. Panel B. Alignment of *Acropora palmata* and *Nematostella* HAP2/GCS1 genomic sequences. Alignment of genomic sequences containing the first coding exon of the HAP2/GCS1 gene from the coral *Acropora palmata* (upper sequence) and the sea anemone *Nematostella vectensis* (lower sequence). Identical amino acids are highlighted in yellow. Intron sequences are in lower case.

The sequences of the genomes of the sponge *Amphimedon queenslandica* and the placozoan *Trichoplax adhaerens* have only recently become available, thus precluding a clear definition in previously published studies [6], [7], [8], [9] of the evolutionary history of the HAP2/GCS1 gene in early diverging metazoan phyla. From the NCBI trace archive, we have identified sequences from a HAP2/GCS1 gene in the genome of the sponge *Amphimedon queenslandica*. Alignment of the sponge sequence with the corresponding sequence from the sea anemone *Nematostella vectensis* shows that the positions of three introns are perfectly conserved between the two genes (Fig. 1A). This result confirms that the sponge gene is orthologous to the HAP2/GCS1 gene. We have also identified a HAP2/GCS1 gene in a third cnidarian, the coral *Acropora palmata* (Fig. 1B). The presence of HAP2/GCS1 genes in hydrozoans and anthozoans indicates that this gene was present in the ancestor of all modern cnidarians [10], [11].

Queries of the assembled genome of *Trichoplax adhaerens* [4] failed to identify a HAP2/GCS1 homologue. These results are most consistent with a HAP2/GCS1 gene being present in the common ancestor of choanoflagellates and metazoans, being maintained in sponges and cnidarians, and being lost in placozoans and most bilaterian lineages. The evolutionary history of the HAP2/GCS1 gene is revealing when compared to the sexual biology of the organisms that do or don't contain it. Choanoflagellates are not known to undergo sexual reproduction, but the presence of a HAP2/GCS1 gene suggests that they may. *Trichoplax* shows genome sequence evidence of having a sexual cycle [5], but a complete sexual cycle has not been demonstrated. Although sperm have not been observed, *Trichoplax* produces what are believed to be eggs and cleavage stage embryos [5].

### HAP2/GCS1-Related Genes in Arthropods

**Figure 2:**
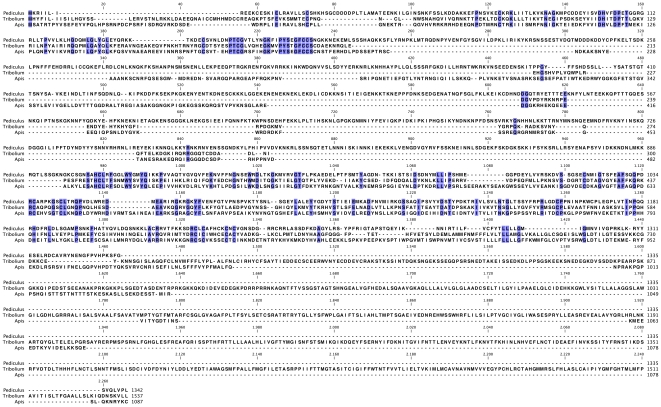
Alignment of arthropod HAP2/GCS1-related protein sequences. Predicted HAP2/GCS1-related protein sequences from arthropods whose genomes have been sequenced and assembled were aligned using T-Coffee. Blue highlighting indicates identical amino acids. The sources of the sequences is as follows: *Pediculus*, Accession Number XP_002429972; *Tribolium*, Accession Number XP_973371; *Apis*, predicted from the *Apis* genome assembly by Liu et al. [9].

**Figure 3:**
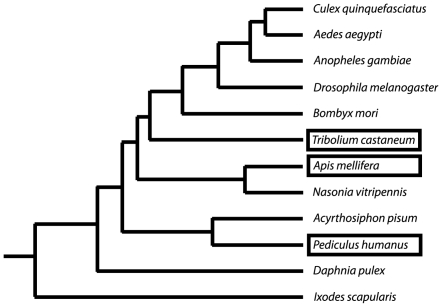
Arthropod phyla containing HAP2/GCS1-related genes. The tree shows the phylogenetic relationships of arthropods for which assembled genomes sequences are available [18], [19]. Boxed organisms contain HAP2/GCS1-related genes.

Liu et al. [9] reported the presence of HAP2/GCS1-related genes in the genomes of two insects, the honeybee *Apis mellifera* and the flour beetle *Tribolium castaneum*. By querying all available assembled arthropod genome sequences, we have identified a HAP2/GCS1-related gene in the genome of a third arthropod, the body louse *Pediculus humanus*. An alignment of the predicted HAP2/GCS1-related protein sequences from *Apis*, *Tribolium*, and *Pediculus* is shown in Fig. 2. In the *Tribolium* gene model, the HAP2/GCS1 sequence is fused to a sequence encoding a polypeptide related to synaptic vesicle protein 2B [12]. Whether this model is correct has not been tested experimentally. It is possible that the model is an artifactual fusion of sequences from two different genes. The *Pediculus* sequence contains a large insertion that is absent from *Tribolium* and *Apis*. Using the amino acid sequence of the *Pediculus* insertion as a query, we were unable to identify related sequences in any other organism. Fig. 3 shows a tree indicating that HAP2/GCS1-related genes are absent from a number of arthropod genomes. Mapping of the presence/absence of HAP2/GCS1-related genes onto the phylogenetic tree of the available arthropod genomes suggests that HAP2/GCS1-related genes have been secondarily lost multiple times within the arthropod lineage. There are no published expression data for the arthropod HAP2/GCS1-related genes and tblastn queries of dbEST [13] with the protein sequences yielded no hits for *Pediculus* or *Apis*. Two ESTs from *Tribolium* larvae were found in dbEST. Both of these ESTs contain only the sequences related to synaptic vesicle protein 2B.

### HAP2/GCS1 Is Expressed Exclusively in Spermatogenic Cells in *Hydra*

**Figure 4:**
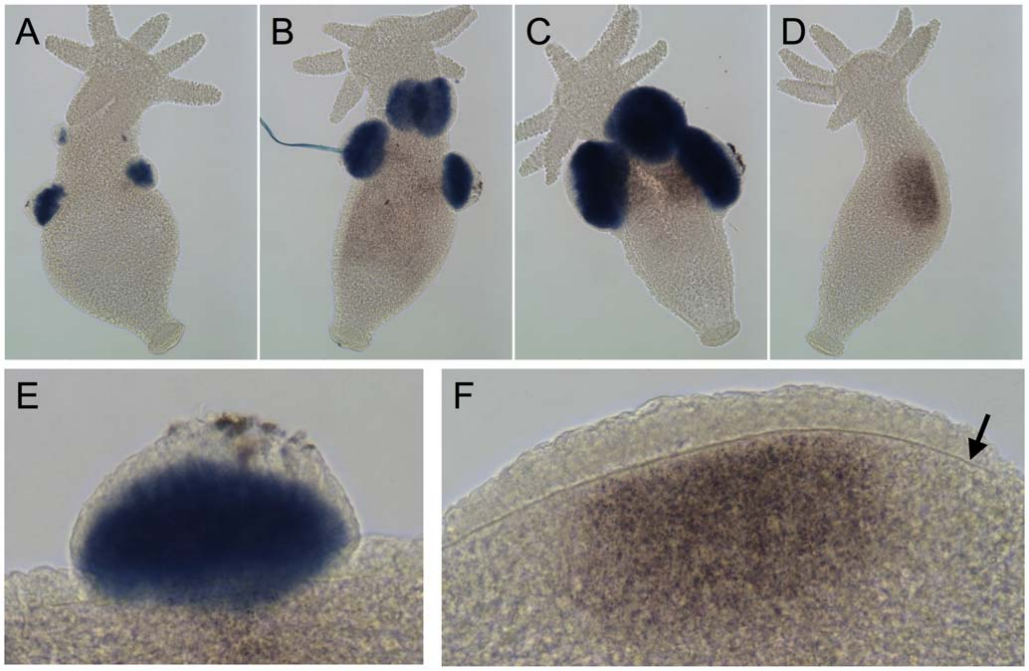
Expression of the HAP2/GCS1 gene in sexual *Hydra*. Whole-mount in situ hybridization was carried out on sexual *Hydra* using a digoxigenin-labeled antisense RNA probe generated from a *Hydra* HAP2/GCS1 cDNA. Panels A–C show sexual male animals, each with multiple testes. Panel D shows a sexual female animal. Panels E and F show, respectively, enlarged views of a testis from the animal in Panel B and the egg-forming region of the animal in Panel D. Panel E shows that the labeled cells are within the lumen of the testis, the location of spermatogenic cells. In Panel F, the egg-forming cells are located within interstices between the ectodermal epithelial cells. The pigment in Panel F is contained in endodermal cells underlying the egg-forming region. This pigment accumulates during oogenesis [20]. The arrow in Panel F indicates the location of the mesoglea, the basement membrane that separates the endodermal epithelium from the ectodermal epithelium.

To determine whether HAP2/GCS1 might play a role in sexual reproduction in metazoans, we examined expression of this gene in sexual polyps of the cnidarian *Hydra*. We found no HAP2/GCS1 sequences among the 152,920 ESTs from asexual *Hydra* and the 5786 ESTs from *Hydra* producing eggs in GenBank. We found one HAP2/GCS1 sequence among the 5619 ESTs in GenBank from *Hydra* with testes. These data suggested that HAP2/GCS1 expression in *Hydra* is restricted to animals producing sperm. To test this hypothesis and to determine the cell type(s) expressing HAP2/GCS1, we examined HAP2/GCS1 expression in sexual *Hydra* by in situ hybridization [14]. We used the AEP strain of *Hydra vulgaris*, which produces both sperm and eggs routinely in the laboratory. The HAP2/GCS1 gene is expressed in the spermatogenic cells in the testis but not in the cells forming an egg (Fig. 4). Expression of the HAP2/GCS1 gene was not detected in the somatic cells of either male or female polyps. This result is in keeping with findings in plants and unicellular eukaryotes, in which HAP2/GCS1 expression is found exclusively or predominantly in male gametes [6], [7], [8], [9], [15].

## Discussion

Our results support a scenario in which HAP2/GCS1-mediated sperm-egg fusion was established in an ancestor of modern plants, protists, and animals, maintained through the lineages leading to modern sponges and cnidarians, and lost from placozoans and most bilaterians. The absence of HAP2/GCS1 from *Trichoplax* suggests that this organism has a derived mechanism of sexual reproductive cycle compared to other basal metazoans.

The distribution of sequences encoding HAP2/GCS1-related proteins in arthropods is unusual. This distribution requires multiple losses of a HAP2/GCS1-related gene during radiation of the arthropods (e.g. loss in the line leading to *Nasonia* following divergence from the line leading to *Apis*). Genome sequences from additional arthropods and expression data for the genes in *Pediculus*, *Apis*, and *Tribolium* are needed to clarify the history and function of HAP2/GCS1-related genes in these organisms. At the least, we can conclude that the history of HAP2/GCS1-related genes in arthropods is complex.

Our results are consistent with the hypothesis that the ancestor of all modern plants, protists, and animals used HAP2/GCS1 as part of its sexual reproduction machinery and suggest that the biochemistry of gamete fusion is extraordinarily ancient. Confirmation of this hypothesis will require functional tests involving the HAP2/GCS1 genes in sponges and cnidarians. A particularly interesting implication of our findings is that the regulatory machinery for controlling expression of the HAP2/GCS1 gene in the male gamete was established in the ancestor of all modern eukaryotes. Investigation of the components of this regulatory machinery in diverse organisms, particularly transcription factors that bind to the HAP2/GCS1 promoter, should be informative with regard to how gametogenesis evolved in eukaryotes.

## Materials and Methods

### Identification of *Amphimedon queenslandica* and *Acropora palmata* HAP2/GCS1 Gene Sequences

HAP2/GCS1-encoding sequences in the *Amphimedon queenslandica* and *Acropora palmata* genomes were identified by a tblastn query against sequences in the Compagen database [16]. This database contains all of the sequence reads from the *Amphimedon* genome project, which is being carried out by the Department of Energy Joint Genome Institute. The *Amphimedon* sequence reads in the Compagen database were obtained from the NCBI trace archive. The *Acropora palmata* sequences in Compagen are from survey genome sequencing carried out at the Genome Sequencing Center at Washington University (http://genome.wustl.edu/genome.cgi?GENOME=Acropora%20palmata), and were also obtained from the NCBI trace archive.

### Sequence Alignment

HAP2/GCS1 amino acid sequences from *Apis*, *Pediculus*, and *Tribolium* were aligned using T-Coffee [17] as implemented on the server at the Swiss Institute of Bioinformatics (http://tcoffee.vital-it.ch/cgi-bin/Tcoffee/tcoffee_cgi/index.cgi). The alignment was saved as a Phylip file and the graphic was generated using Version 6.2 of CLC Sequence Viewer (www.clcbio.com).

### In Situ Hybridization

The insert of the *Hydra* HAP2/GCS1 clone from the male polyp cDNA library [9] was amplified using primers corresponding to the T7 and SP6 promoters in the plasmid. Digoxigenin-labeled antisense and sense probes were prepared by in vitro transcription of the amplified cDNA insert using the DIG RNA Labeling Kit (Roche) and T7 (sense) and SP6 (antisense) RNA polymerases. RNA was precipitated from the transcription reaction mixture using a LiCl precipitation solution (Ambion). Whole-mount in situ hybridization was carried out essentially as described in Bode et al. [14]. *Hydra* polyps were mounted in Euparal (Carolina Biological Supply Company) prior to being photographed. Hybridization with a sense probe gave no signal.
